# Pediatric injuries and poisonings associated with detergent packets: results from the Canadian Hospitals Injury Reporting and Prevention Program (CHIRPP), 2011–2023

**DOI:** 10.1186/s40621-024-00513-5

**Published:** 2024-07-11

**Authors:** Sarah Zutrauen, James Cheesman, Steven R. McFaull

**Affiliations:** Centre for Surveillance and Applied Research, Health Promotion and Chronic Disease Prevention Branch, Public Health Agency of Canada, Ottawa, ON Canada

**Keywords:** Detergent packet, Laundry pod, Dishwasher tablet, Poisoning, Ingestion, Eye injury, Injury, Emergency department

## Abstract

**Background:**

Detergent packets are common household products; however, they pose a risk of injuries and poisonings, especially among children. This study examined the epidemiological characteristics of pediatric injuries and poisonings related to all types of detergent packets in Canada using emergency department (ED) data from the Canadian Hospitals Injury Reporting and Prevention Program (CHIRPP) database.

**Methods:**

The CHIRPP database was searched for ED visit records for injuries and poisonings related to all types of detergent packets between April 1, 2011 and October 12, 2023 (*N* = 2,021,814) using variable codes and narratives. Data for individuals aged 17 years and younger were analyzed descriptively. Temporal trends in the number of detergent packet-related injuries and poisonings per 100,000 CHIRPP cases were assessed using Joinpoint regression and annual percent change (APC). A proportion ratio and 95% confidence intervals (CI) were calculated to compare the proportion of detergent packet-related cases in CHIRPP during two 34-months periods, pre-COVID-19 pandemic and after the beginning of the pandemic.

**Results:**

There were 904 detergent packet-related cases among children and youth aged 17 years and younger identified in CHIRPP between April 1, 2011 and October 12, 2023, representing 59.9 cases per 100,000 CHIRPP cases. The majority (86.5%) of cases were among children aged 4 years and younger. Poisonings (58.8%) and eye injuries (30.6%) were the most frequent primary diagnoses. Unintentional ingestion (56.9%) and squeezing/breaking a detergent packet (32.3%) were the most frequent exposure mechanisms. Sixty-five patients (7.2%) were admitted to hospital. The number of detergent packet-related cases per 100,000 CHIRPP cases increased by 5.0% (95% CI 0.8, 10.2) annually between 2012 and 2022. The number of detergent packet-related poisonings per 100,000 CHIRPP cases decreased by 15.3% (95% CI − 22.3, − 10.6) annually between 2015 and 2022, whereas eye injuries showed an average annual percent increase of 16.6% (95% CI 11.2, 23.0) between 2012 and 2022. The proportion of detergent packet-related cases in CHIRPP after the beginning of the pandemic (79.9/100,000 CHIRPP cases) was 1.43 (95% CI 1.20, 1.71) times greater than pre-pandemic (55.7/100,000 CHIRPP cases).

**Conclusions:**

Detergent packet-related injuries and poisonings are a persisting issue. Continued surveillance and prevention efforts are needed to reduce detergent packet-related injuries and poisonings in Canada, particularly among children and youth.

**Supplementary Information:**

The online version contains supplementary material available at 10.1186/s40621-024-00513-5.

## Background

Single-use detergent packets have been available in Europe since 2001 (Mathew et al. 2010) and were introduced into the North American market around 2010 (Centers for Disease Control and Prevention (CDC) 2012; Bonney et al. 2013). These detergent packets, also known as detergent pods, capsules, sachets, tablets or sacs, contain a concentrated detergent often encapsulated within a water-soluble membrane and are advertised as a more compact and convenient substitute for the traditional laundry or dishwasher detergent (Valdez et al. 2014; Sebastian et al. 2014; Forrester 2013). However, the appearance of detergent packets may be particularly attractive for young children as it could easily be mistaken for a toy or food (Glenn 2015).

Ingestion is the most common route of detergent packet exposure (Bonney et al. 2013; Day et al. 2019a), accounting for approximately 80% of poison control centre calls in a US study (Valdez et al. 2014) and poses a serious poisoning risk, especially to children younger than 6 years of age. Studies suggest that the outcomes of laundry detergent packet ingestion tend to be more serious than those involving traditional laundry detergents (Davis et al. 2016; Swain et al. 2016). Ocular exposures have also been well documented in the literature (Mathew et al. 2010; Lasnier et al. 2013; Haring et al. 2017; Breazzano et al. 2018). Dermal exposure to detergent packets, though being less common, can result in skin irritation, rashes and/or chemical burns (Williams et al. 2012; Russell et al. 2014).

Poisonings, eye injuries and skin irritation caused by detergent packet-related exposures have been reported in Europe (Day et al. 2019b; Villa et al. 2014; Settimi et al. 2018). Shortly after detergent packets were introduced into North America, there were reports of related poisonings and injuries among children (Centers for Disease Control and Prevention (CDC) 2012). Between January 2014 and December 2022, over 114,000 laundry detergent packet exposures among children younger than 6 years of age were reported to the US National Poison Data System (NPDS) (Zhang et al. 2023). Furthermore, a US study which analyzed emergency department (ED) data from the National Electronic Injury Surveillance System (NEISS) between 2016 and 2020 found an estimated 13,176 detergent packet-related oral-aerodigestive injuries and ingestion poisonings, as well as an estimated 8,654 detergent packet-related ocular injuries among individuals younger than 18 years of age (Wiener et al. 2023).

In Canada, a retrospective review of three EDs identified 40 cases of detergent packet exposures between 2009 and 2014 (Rosenfield et al. 2018). Further, between April 2011 and March 2014, an analysis identified 53 cases related to laundry detergent packets presenting to select EDs across Canada (Do and Cheesman 2015). Results from a Canadian survey of paediatricians in 2014 revealed 54 cases of children who were injured following exposure to detergent packets in a one-year period (Do et al. 2015). Overall, there are limited recent studies related to detergent packet exposures in Canada. Furthermore, there are few studies (Davis et al. 2016; Do et al. 2015) to our knowledge that examine other types of detergent packets, such as dishwasher detergent packets, as much of the literature is focused on laundry detergent packets. Though the composition of various detergent packets (e.g., laundry compared to dishwasher) might differ, the concept of the product usages are comparable and the implications for injury prevention may be similar.

To fill this data gap, this study aims to describe the epidemiological characteristics of pediatric ED presentations for injuries and poisonings related to all types of detergent packets in Canada between April 2011 and October 2023 using data from the Canadian Hospitals Injury Reporting and Prevention Program (CHIRPP) database.

## Methods

### Data source

The Canadian Hospitals Injury Reporting and Prevention Program (CHIRPP) is an ED-based injury and poisoning sentinel surveillance system, currently operating in the EDs of 11 pediatric hospitals and 9 general hospitals across Canada (Public Health Agency of Canada 2022). CHIRPP was established in 1990, and the system transitioned to an electronic format in 2011 (Crain et al. 2016). CHIRPP was established in 1990, and the system transitioned to an electronic format in 2011 (Crain et al. 2016). CHIRPP was not designed to produce nationally representative incidence estimates of specific types of injuries and poisonings. CHIRPP collects detailed data of “pre-event” injury information, including details regarding how the injury or poisoning occurred. During their visit to the ED of a participating CHIRPP hospital, the patient (or the accompanying caregiver) is asked to complete an injury reporting form detailing the circumstances of the injury or poisoning event. These records are supplemented by clinical information input by the attending physician or hospital staff. The details are entered into the CHIRPP database, and data coders extract additional information found in patients’ narratives of what happened.

### Case extraction

For this cross-sectional study, we searched the CHIRPP database for cases (all ages) of injuries and poisonings related to all types of detergent packets occurring between April 1, 2011 and October 12, 2023 (*N* = 2,021,814). The CHIRPP factor codes for dishwashing liquid (421F), dishwasher detergents (422F), laundry soaps or detergents (423F), detergents not further specified (424F), combined with a text search with English and French terms such as “detergent”, “packet”, “tide”, or “pod” of the patient narrative (description of the injury event), substance ID and product fields were used to identify potential cases. This search identified 967 potential detergent packet-related cases. To ensure accuracy of the case selection, the search query was followed by manual identification and confirmation of cases by reading through each narrative. Additionally, further details were coded from manual review of the narratives, including the type of detergent packet, the exposure mechanism and the context surrounding the injury and/or poisoning event. Cases where it was unclear whether the injury or poisoning event was related to a detergent packet were excluded (*n* = 41). Detergent packet-related cases among individuals aged 18 years and older (*n* = 22) were excluded from the present analysis.

### Statistical analysis

Descriptive analyses were conducted to examine the distribution of characteristics of pediatric injury and poisoning cases related to all types of detergent packets, overall and stratified by sex over the entire study period (April 1, 2011–October 12, 2023). Variables of interest included patient age, type of detergent packet involved, primary nature of injury, location and area where the patient was at the time of injury or poisoning, treatment received and intent. Unintentional injuries were defined as any injury that had no intention to harm or that was not caused on purpose (Chen et al. 2013). Intentional injuries were defined as a deliberate act of harm to oneself (e.g., self-harm/suicide) or another person (e.g., assault) (Yao et al. 2020). The primary nature of injury variable is composed of CHIRPP codes for the clinical injury or poisoning diagnosis recorded by the attending physician in the emergency department. Counts and example narratives regarding the context surrounding the detergent packet-related injury or poisoning as well as the exposure mechanism are also presented.

To quantify CHIRPP’s capture of detergent packet-related injury and poisoning cases, the data were normalized to the total number of cases in CHIRPP for the given year, sex, age group and primary nature of injury, and presented as the number of detergent packet-related cases per 100,000 CHIRPP cases (McFaull et al. 2012). We evaluated temporal trends in the number of detergent packet-related cases per 100,000 CHIRPP cases between 2012 and 2022 using Joinpoint regression (Joinpoint Regression Program 2023). Years 2011 (*n* = 2) and 2023 (*n* = 37) were excluded from the time trend analysis as they did not include all months of the year. The Joinpoint regression detects inflection points, outputs the average annual percent change (AAPC), calculates whether the annual percent change (APC) of identified segments are significantly different from zero (*α* = 0.05) and produces 95% Confidence Intervals (CI) (Kim et al. 2000). Findings were cross-validated by applying SAS complex survey software to the record-level data.

A proportion ratio and 95% CIs were calculated to compare the proportion of detergent packet-related cases in CHIRPP pre-COVID-19 pandemic (March 11, 2017 to December 31, 2019) to the proportion after the beginning of the pandemic (March 11, 2020 to December 31, 2022). The beginning of the pandemic period is defined as March 11, 2020, which coincides with the World Health Organization’s statement declaring the novel COVID-19 outbreak a global pandemic (World Health Organization (WHO) 2020). Each period comprised a time span of approximately 34 months.

All analyses were performed using PC SAS® software version 9.4 (SAS Institute Inc., Cary, NC, USA) and Joinpoint Regression Program version 5.0.2 (Joinpoint Regression Program 2023).

## Results

Overall, between April 1, 2011 and October 12, 2023, there were 904 injury and poisoning cases associated with all types of detergent packets (59.9/100,000 CHIRPP cases) among children and youth aged 17 years or younger (0–215 months) presenting to EDs participating in CHIRPP. Table 1 shows the distribution of selected characteristics. Males accounted for 51.9% of the ED visits, and 86.5% of cases occurred among children aged 4 years or younger. The mean and median age was 2.7 years (SD = 3.0) and 2.0 years (IQR = 2.0), respectively. Poisonings (58.8%) and eye injuries (30.6%) were the most frequent primary injury diagnoses. There were 20 burn injuries (excluding burns of the eye or internal caustic burns), of which 55.0% were burns to the face. Fifty-four cases had multiple injuries, of these the most common co-occurring injuries were poisoning and eye injury (50.0%), multiple eye injuries (18.5%), followed by eye injury and burn (9.3%) (Supplementary file 1). Laundry detergent packets were related to 81.0% of cases, of which 49.5% were among children aged 2–4 years, 55.3% resulted in poisoning and 35.0% in eye injuries (Supplementary file 2). Dishwasher detergent packets were related to 15.2% of cases, of which 78.1% were among children younger than 2 years of age and 75.9% resulted in poisoning (Supplementary file 2). Where location information was specified (*n* = 771), 97.3% occurred in a private home. Of the 65 patients who were hospitalized, 69.2% were males, 61.5% were among children younger than 2 years of age and 83.1% were due to unintentional ingestion (Supplementary File 3). Most detergent packet-related injuries and poisonings were unintentional in nature (97.6%). However, there were 22 cases (2.4%) of intentional self-harm, all of which were among youth aged 10–17 years and occurred via ingestion of a laundry detergent packet. More specifically, 77.3% of intentional cases were among females and 72.7% occurred between 2020 and 2023 (Supplementary file 4). There were no fatal cases reported to CHIRPP.

**Table 1:** Characteristics of detergent packet-related injuries and poisonings among children and youth 17 years of age and younger, overall and by sex, CHIRPP, April 1, 2011–October 12, 2023 (*N* = 904)

Characteristic	Total		Males		Females	
	*n*	(column %)	*n*	(column %)	*n*	(column %)
*Age group (years)*						
< 1	91	(10.1)	46	(9.8)	45	(10.3)
1	300	(33.2)	158	(33.7)	142	(32.6)
2–4	391	(43.3)	205	(43.7)	186	(42.8)
5–9	75	(8.3)	45	(9.6)	30	(6.9)
10–17	47	(5.2)	15	(3.2)	32	(7.4)
*Type of detergent packet*						
Laundry	732	(81.0)	385	(82.1)	347	(79.8)
Dishwasher	137	(15.2)	66	(14.1)	71	(16.3)
Toilet	7	(0.8)	4	(0.9)	3	(0.7)
Detergent packet, NFS	28	(3.1)	14	(3.0)	14	(3.2)
*Primary nature of injury*						
Poisoning or toxic effect	532	(58.8)	283	(60.3)	249	(57.2)
Eye injury^a^	277	(30.6)	139	(29.6)	138	(31.7)
Burn or corrosion^b^	20	(2.2)	12	(2.6)	8	(1.8)
Face	11		8		3	
Trunk	3		1		2	
Other body part^c^	6		3		3	
Foreign body in respiratory/alimentary tract	9	(1.0)	5	(1.1)	4	(0.9)
Asphyxia	3	(0.3)	2	(0.4)	1	(0.2)
Internal caustic burn	3	(0.3)	1	(0.2)	2	(0.5)
Superficial injury	3	(0.3)	0	(0.0)	3	(0.7)
Foreign body in ear canal or soft tissue	2	(0.2)	1	(0.2)	1	(0.2)
No injury detected or not specified	55	(6.1)	26	(5.5)	29	(6.7)
*Location*						
Private home	750	(83.0)	382	(81.4)	368	(84.6)
Other specified location^d^	21	(2.3)	13	(15.8)	8	(13.6)
Unspecified location	133	(14.7)	74	(2.8)	59	(1.8)
*Area*						
Laundry room	170	(18.8)	91	(19.4)	79	(18.2)
Kitchen	125	(13.8)	63	(13.4)	62	(14.3)
Living room	34	(3.8)	16	(3.4)	18	(4.1)
Bathroom	28	(3.1)	14	(3.0)	14	(3.2)
Basement, cellar	19	(2.1)	11	(2.3)	8	(1.8)
Other specified area^e^	27	(3.0)	10	(2.1)	17	(3.9)
Unspecified area	501	(55.4)	264	(56.3)	237	(54.5)
*Treatment*						
Left without being seen	24	(2.7)	13	(2.8)	11	(2.5)
Advice only	188	(20.8)	79	(16.8)	109	(25.1)
Treated in ED, follow-up as needed	266	(29.4)	133	(28.4)	133	(30.6)
Observation in ED, follow-up required or as needed	165	(18.3)	92	(19.6)	73	(16.8)
Treated in ED, follow-up required	196	(21.7)	107	(22.8)	89	(20.5)
Admitted to hospital	65	(7.2)	45	(9.6)	20	(4.6)
*Intent*						
Unintentional	882	(97.6)	464	(98.9)	418	(96.1)
Intentional	22	(2.4)	5	(1.1)	17	(3.9)
*Total*	904	(100.0)	469	(51.9)^f^	435	(48.1)^f^

*CHIRPP* Canadian Hospitals Injury Reporting and Prevention Program; ED, emergency department; NFS, not further specified

^a^"Eye injury" category includes globe only (including eye burn/corrosion) and foreign body in external eye

^b^"Burn or corrosion" category excludes eye injury and internal caustic burn

^c^"Other body part" includes forearm, internal mouth/neck, and injuries to multiple body parts

^d^"Other specified location" category includes own cottage/cabin, shop/shopping centre, institutional home, other institution, community centre, laundromat, hotel, construction site and road

^e^"Other specified area" category includes bedroom, classroom, hall/foyer, storage room and driveway

^f^Represents a row percentage

Table 2 shows the detergent packet-related ED visit counts and frequency per 100,000 CHIRPP cases by age, sex and pandemic period. Once normalized to all CHIRPP cases of the same age group, one-year-olds were the most frequent at 241.8/100,000 CHIRPP cases. Although males accounted for 51.9% of the ED visits for detergent packet-related injuries and poisonings, females consistently displayed higher proportions of detergent packet-related injuries and poisonings relative to all CHIRPP cases, except for the 5–9 years age group. The overall proportion of detergent packet-related cases in CHIRPP after the beginning of the COVID-19 pandemic (79.9/100,000 CHIRPP cases) was 1.43 (95% CI 1.20, 1.71) times greater than pre-pandemic (55.7/100,000 CHIRPP cases).

**Table 2:** Detergent packet-related ED visit counts and frequency per 100,000 CHIRPP cases, children and youth 17 years of age and younger, by sex, age and pandemic period, CHIRPP, April 1, 2011–October 12, 2023 (*N* = 904)

Characteristic		Number of cases	Number per 100,000 CHIRPP cases^a^
Female	*Age group (years)*		
	0–17	435	65.3
	< 1	45	126.5
	1	142	252.6
	2–4	186	143.8
	5–9	30	19.5
	10–17	32	11.0
	*Pandemic period*^*b*^		
	Pre-pandemic	108	64.1
	After the beginning of the pandemic	132	79.9
Male	*Age group (years)*		
	0–17	469	55.7
	< 1	46	114.7
	1	158	232.9
	2–4	205	122.4
	5–9	45	22.3
	10–17	15	4.1
	*Pandemic period*^*b*^		
	Pre-pandemic	104	49.1
	After the beginning of the pandemic	154	80.0
Total	*Age group (years)*		
	0–17	904	59.9
	< 1	91	120.3
	1	300	241.8
	2–4	391	131.7
	5–9	75	21.1
	10–17	47	7.2
	*Pandemic period*^*b*^		
	Pre-pandemic	212	55.7
	After the beginning of the pandemic	286	79.9

*CHIRPP* Canadian Hospitals Injury Reporting and Prevention Program

aExpressed as a normalized frequency per 100,000 CHIRPP cases in the given sex, age group and time period

^b^Pre-pandemic period is defined as cases which occurred between March 11, 2017 and December 31, 2019. After the beginning of the pandemic period is defined as cases which occurred between March 11, 2020 and December 31, 2022

Table 3 shows the exposure mechanism and context leading to the detergent packet-related ED visit, as well as sample CHIRPP injury and poisoning event narratives. Overall, unintentional ingestion of a detergent packet was the leading exposure mechanism (56.9%), followed by squeezing/breaking a detergent packet (32.3%). Among children aged 0–4 years, unintentional ingestion was most frequent (63.6%), whereas among 5–9 year-olds, squeezing/breaking a detergent packet was most frequent (73.3%) (Supplementary file 5). Among 10–17 year-olds, the leading mechanisms were squeezing/breaking a detergent packet (48.9%) followed by intentional ingestion (46.8%) (Supplementary file 5). No cases referencing the viral social media trend (Quail 2018) of biting or ingesting a detergent packet were identified in the CHIRPP data. Where the context was reported (*n* = 491), the patient was playing in 85.5% of cases at the time of injury, such as while playing with a friend or sibling, playing in the cupboard or under the sink, or climbing onto a table, counter, or shelf. Forty-nine patients were doing (or helping with) laundry/cleaning and 22 involved self-harm or suicide attempt.

**Table 3:** Exposure mechanism and context leading to an emergency department visit associated with detergent packet-related injuries and poisonings among children and youth 17 years of age and younger, CHIRPP, April 1, 2011–October 12, 2023 (*N* = 904)

	Number of cases	Example narrative
*Exposure mechanism*		
Unintentional ingestion	514	Playing. Ingested [brand name] dishwasher pod. Vomit × 2
Squeezed/broke DP and contents got into eye/onto face or body	292	Squeezed laundry pod and it broke, laundry pod burst in face, chemical exposure to eyes
Bit into DP and contents got into eye/onto face or body	36	Patient accidentally bit into a [brand name] laundry pod no ingestion but in squirted into L eye. Had L eye flushed. Eye still red and puffy with sticky yellow discharge and eye seems more sensitive to light
DP contents on hands and touched eye/face	28	Crushed a [brand name] laundry pod and then rubbed her eye with her hands
Intentional ingestion	22	^a^
DP contents on bedding/clothing	5	Mother had [brand name] laundry pod in diaper bag and found it had exploded. Later changed baby with diaper that had been in same bag. Chemical burns to baby's lower back and thighs
Unknown or not further specified	7	Playing with [brand name] laundry pod
*Context*		
Playing	420	Playing at home and mom found patient eating dishwasher detergent pod
Playing, with sibling/friend	31	Playing with her older sister when she was given a dishwasher pod and bit into it. Very small amount ingested
Playing, DP left out/in reach	27	Bag of [brand name] laundry pods dropped floor baby got hold of one and bit into it
Playing, got into cupboard	21	Playing at home, lock left open under kitchen sink and hold of dishwasher tab and ate some
Playing, reached DP on table/counter/shelf	14	Patient pushed his chair up to a counter, got a hold of a [brand name] laundry detergent pod, popped pod, and got pod laundry detergent on his face and in his eyes
Playing, NFS	327	Playing with [brand name] laundry pod, sprayed self in eye
Doing laundry/cleaning (including helping parent)	49	Helping mom with washing and took the [brand name] laundry pod and decided to put it in her mouth. It exploded in her face
Self-harm/suicide attempt	22	^a^
Not specified	413	Bit on the detergent pod, not further specified

*CHIRPP* Canadian Hospitals Injury Reporting and Prevention Program, *DP* detergent packet, *NFS* not further specified

^a^The example narrative has been suppressed due to the sensitive nature of the topic

**Fig. 1:**
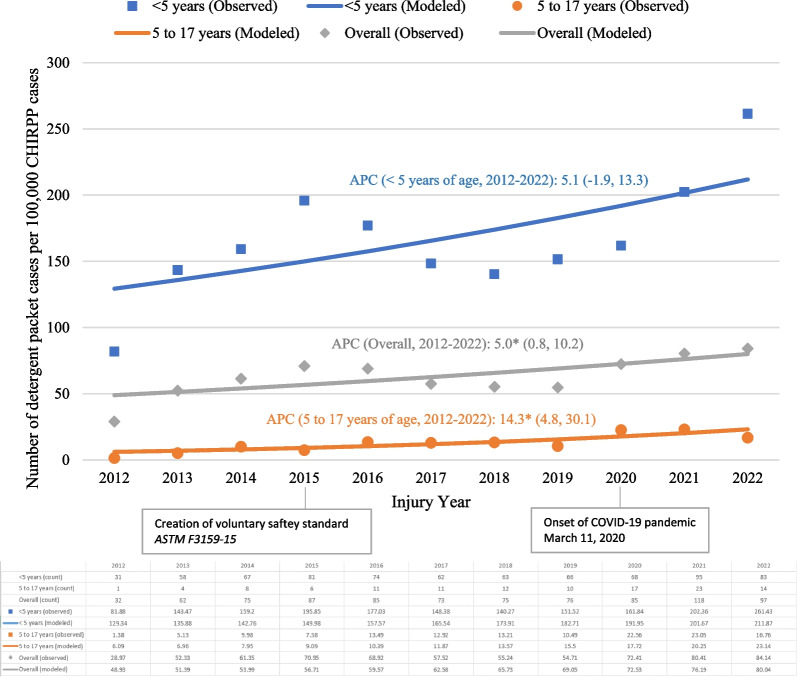
Number of detergent packet-related ED visit cases per 100,000 CHIRPP cases, children and youth 17 years of age and younger, overall by age group, January 1, 2012 to December 31, 2022 (*N* = 865). Abbreviations: APC, annual percent change; CHIRPP, Canadian Hospitals Injury Reporting and Prevention Program. *Note* Expressed as a normalized frequency per 100,000 CHIRPP cases in the given year and age group. *Represents significantly different from zero at the *α* = 0.05 level

Figure 1 shows the observed and modeled results from the Joinpoint regression for the number of detergent packet-related cases per 100,000 CHIRPP cases between 2012 and 2022, overall and by age group. Overall, a significant annual increase of 5.0% was observed between 2012 and 2022 (95% CI 0.8, 10.2). A non-significant annual increase of 5.1% (95% CI − 1.9, 13.3) was observed among children younger than 5 years of age, and a significant annual increase of 14.3% (95% CI 4.8, 30.1) was observed among children and youth aged 5–17 years between 2012 and 2022.

**Fig. 2:**
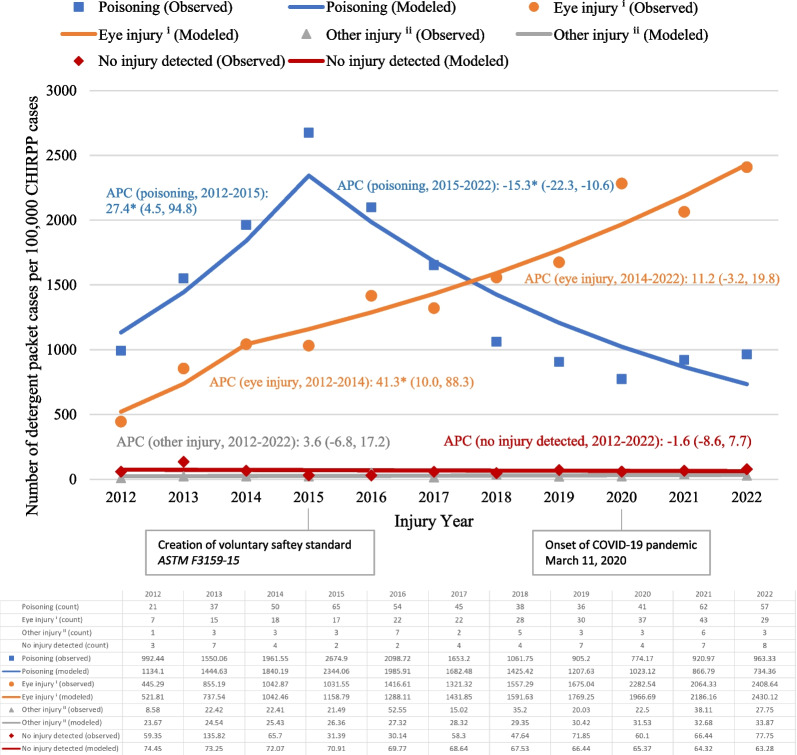
Number of detergent packet-related ED visit cases per 100,000 CHIRPP cases, children and youth 17 years of age and younger, by primary nature of injury, January 1, 2012 to December 31, 2022 (*N* = 865). Abbreviations: APC, annual percent change; CHIRPP, Canadian Hospitals Injury Reporting and Prevention Program. *Note* Expressed as a normalized frequency per 100,000 CHIRPP cases in the given year and primary nature of injury category. *Represents significantly different from zero at the *α* = 0.05 level. ⁱEye injury includes injury to the globe and foreign body in external eye. ⁱⁱ"Other injury" includes: superficial, burn or corrosion, asphyxia, internal caustic burn, and foreign body in respiratory tract, alimentary tract, ear canal or soft tissue

Figure 2 shows the results from the Joinpoint regression, by primary nature of injury. The number of detergent packet-related poisonings per 100,000 CHIRPP cases significantly increased between 2012 and 2015 (APC = 27.4, 95% CI 4.5, 94.8), followed by a significant decrease between 2015 and 2022 (APC = − 15.3, 95% CI − 22.3, − 10.6), yielding a non-significant average annual decrease between 2012 and 2022 of 4.3% (AAPC = − 4.3%, 95% CI − 8.9, 2.4). The number of detergent packet-related eye injuries per 100,000 CHIRPP cases showed a significant average annual increase between 2012 and 2022 (AAPC = 16.6, 95% CI 11.2, 23.0).

## Discussion

A total of 904 ED visits related to all types of detergent packets among children and youth 17 years of age and younger were identified in CHIRPP between April 1, 2011 and October 12, 2023. In line with previous studies (Valdez et al. 2014; Settimi et al. 2018), approximately half of the detergent packet cases identified in our study were among males. Though most published studies focus on laundry detergent packet exposures only, our study was consistent with the literature (Centers for Disease Control and Prevention (CDC) 2012; Williams et al. 2014; Vohra et al. 2020; Rocka et al. 2021) such that children younger than 5 years of age accounted for a large majority of ED visits related to all types of detergent packets reported to CHIRPP. The appearance of detergent packets may be particularly attractive for young children as it could easily be mistaken for a toy or food (Glenn 2015). Although detergent packet-related injuries and poisonings among adults were excluded from the present analysis, there have been reports of detergent packet exposure among older adults, including adults with dementia (Tark 2020). A US study using data from the NPDS found that detergent packet exposures among older adults increased over 400% between 2012 and 2020 (Aldy et al. 2022). Continued surveillance among this disproportionately affected population is needed.

Our analysis identified that, similar to other studies (Valdez et al. 2014; Swain et al. 2016; Settimi et al. 2018; Do and Cheesman 2015; Williams et al. 2014), the most common mechanism of detergent packet exposure reported to CHIRPP was unintentional ingestion (especially among children 4 years of age and younger), followed by squeezing/breaking the detergent packet (especially among children aged 5–9 years). The developmental stage among young children may put them at higher risk for ingestion or eye exposure due to their exploratory interest in their senses (Glenn 2015; Richmond et al. 2020). Furthermore, the membrane surrounding the detergent is designed to dissolve when in contact with water, creating a risk of exposure if handled with wet hands or if placed in the mouth (National Poison Data System, America's Poison Centers 2024). Where the context was reported, most patients accessed the detergent packet while playing, such as while playing in a cupboard or under the sink, climbing onto a table, counter or shelf, or because a detergent packet was left out/within reach. These findings emphasize the importance of the responsibility of caregivers to ensure a child-safe environment and safe storage practices for all types of detergent packets to prevent access by children. This includes storing detergent packets up and out of sight of children and in a locked cabinet; and when the detergent packets are in use, caregivers should not leave the product accessible to children (Davis et al. 2016).

Sixty-five patients were admitted to the hospital for an injury or poisoning associated with a detergent packet over the study period. Over three-quarters were admitted for an unintentional ingestion and over half were among those younger than 2 years of age. This finding underscores the potential for serious toxicity following ingestion of a detergent packet, especially among young children (Schneir et al. 2013).

Intentional detergent packet ingestion was identified in 22 cases in CHIRPP among youth aged 10–17 years. Despite the small sample size of intentional ingestion cases, this finding has serious implications as all 22 cases involved the intention to self-harm including suicide attempt. The majority of intentional cases occurred among females and almost three-quarters occurred between 2020 and 2023. Self-harm and suicide are recognized as major public health concerns among adolescents (Hawton et al. 2012), and emerging evidence suggests that the COVID-19 pandemic may negatively impact the mental health of children and youth (Kauhanen et al. 2023). Recent studies have found that intentional pediatric poisoning ED presentations have increased during the COVID-19 pandemic, and disproportionately affected adolescent females (Gatenby et al. 2023; Zhang et al. 2022; Park et al. 2022; Azkunaga et al. 2023). These findings emphasize the need for poisoning prevention efforts and mental health supports in the youth population, particularly for females.

The initial increase in detergent packet-related ED visits in CHIRPP aligns with the introduction of the products into the North American market (Bonney et al. 2013). When stratified by primary nature of injury, the number of detergent packet-related poisonings per 100,000 CHIRPP cases peaked in 2015, whereas eye injuries increased between 2012 and 2022. This finding is consistent with an analysis of the US NPDS, which identified that the annual number of ingestion exposures to detergent packets among children younger than 6 years of age peaked in 2015, whereas the number of ocular exposures increased steadily between 2012 and 2017 but at a lower rate (Gaw et al. 2019). Reynolds et al. (2020) also found that ocular exposures reported to the NPDS increased between 2012 and 2018. This result was also seen in the NEISS data, where the estimated number of ocular exposures approximately doubled from years 2012 to 2019 (Tark 2020). The decline in detergent packet-related poisonings may be in part due to increased awareness of the risks of detergent packet ingestion as well as various preventive efforts and safety standards implemented around the same time (Gaw et al. 2019; Hanway and Rodgers 2020; Rodgers 2022). In 2015, a voluntary safety standard for liquid laundry packets (ASTM F3159-15e1) was published by the American Society for Testing and Materials (ASTM) International with the aim of reducing unintentional exposures to the contents of the packets, especially to children (ASTM International 2015). The voluntary safety standard applies exclusively to household liquid laundry detergent packets, and calls for child-resistant packaging, opaque containers, clearer health and warning labels and compression and taste/dissolution properties of the packets (Hanway and Rodgers 2020). Notably, the voluntary standard permits manufacturers to meet the requirement for child-resistant packaging in six different ways (Gaw et al. 2019). Though the voluntary ASTM safety standard was implemented in 2015, some manufacturers began making safety changes in 2013 (Reynolds et al. 2020). A market survey conducted by Health Canada in 2018 indicated that the vast majority of products available on the market were respecting the voluntary safety standard (Health Canada 2021). Health Canada issued an advisory (2013) in 2012 regarding liquid laundry detergent packets and has also disseminated information via a webpage (Health Canada 2023) and social media messaging that educate people in Canada about the hazards posed by laundry detergent packets as well as recommendations regarding the safe use of detergent packets. Additionally, Health Canada has communicated to regulated parties that laundry detergent packets are a hazard of concern (2024) and that regulated parties should take the measures necessary to mitigate the hazards identified. Health Canada continually monitors incidents involving liquid laundry detergent packets and actively participates on the subcommittee (ASTM International 2024) responsible for maintaining the ASTM safety standard.

Recent studies examining the impact of the voluntary safety standard have suggested that the standard, in combination with industry and public health educational campaigns, has contributed to a decline in number and severity of unintentional laundry detergent packet exposures among children in the US (Zhang et al. 2023; Gaw et al. 2019; Reynolds et al. 2020; Hanway and Rodgers 2020). A report using emergency department data from the NEISS found that the population adjusted injury rates for laundry packet exposures among children younger than 6 years of age decreased between the pre and post safety standard implementation periods, but the differences were not statistically significant (Tark 2020). Another report using poison centre data found that the population based rates of laundry packet exposures among children younger than 6 years of age increased 6.4% from baseline (July 2012–June 2013) compared to 2020 (Reynolds 2021). However, these two reports also both found decreases in the sales-adjusted rates of laundry packet exposures from pre to post voluntary safety standard implementation (Tark 2020; Reynolds 2021). Notably, Reynolds et al. (2020) used Nielson sales data for laundry packets and found that units of laundry detergent packets sold increased three-fold between 2012 and 2018. Future work focusing on examining the impact of the ASTM voluntary safety standard for liquid laundry packets in the Canadian context is needed. Incorporating the use of Canadian sales data may be particularly useful to contextualize the laundry detergent packet exposures in terms of the availability of the product in Canada.

Our analysis showed that the number of detergent packet-related injuries and poisonings per 100,000 CHIRPP cases was higher after the beginning of the pandemic compared to the pre-pandemic period. Recent reports have indicated that exposures related to household cleaning products, and some foreign body ingestions, especially among children, increased at the onset of the COVID-19 pandemic (Zhang et al. 2022; Yasseen et al. 2023; Chang et al. 2020; Klein et al. 2022; Neal et al. 2022). In 2020 and 2021, household cleaning products, such as dishwasher detergent, bleach, disinfectant and floor cleaners, were reported as the leading cause of non-drug substance exposures managed by Canadian poison centres (Canadian Association for Poison Centres and Clinical Toxicology 2020, 2021). Gulamhusein and Sabri (2021) reported an increase in ocular exposures to laundry detergent packets during the COVID-19 pandemic at a tertiary ophthalmology center in Canada. During the COVID-19 pandemic, various public health measures including lockdowns and daycare or school closures caused many parents and caregivers to experience changes in schedules, such as balancing work and childcare at home (Fong and Iarocci 2020). US emergency department data from a Consumer Product Safety Commission report in 2021 suggested that injuries related to soaps and detergents increased during the March to September 2020 timeframe, perhaps as consumers may have stayed home and done more house cleaning (United States Consumer Product Safety Commission 2021). Thus, it is possible that children may have had increased opportunities to be exposed to household products like detergent packets while spending more time at home (Gulamhusein and Sabri 2021; Harding 2020). A recent study using data from the Italian Pavia Poison Centre (PPC) during a three-month COVID-19 lockdown period compared to the same months of 2017–2018–2019 found a decrease in exposures to liquid laundry detergent packets among children aged 1–5 years (Giordano et al. 2022). A possible explanation for the differing findings observed could be due to the different pandemic periods used, as our study used a period of approximately 34 months. There could also be differences in the distribution of injuries and poisonings captured through ED visits compared to poison centre calls, where poison centre data may contain a lesser percentage of other injuries such as eye injuries, which increased throughout our study period.

Injuries and poisonings related to detergent packets are a persisting issue in Canada. Greater consumer awareness of injuries and poisonings caused by all types of detergent packets, particularly for eye injuries, is needed to improve safety practices (Williams et al. 2012). Furthermore, improvements in safeguarding detergent packets from children and youth is essential; such as through child-resistant packaging, greater parental supervision when using the products, and safe storage practices like using a locked cabinet for storing all types of detergent packets including laundry, dishwasher, and others. Some researchers have suggested that further reductions in injury and poisonings exposures might be possible through requiring that all liquid laundry detergent packet products undergo a formal testing protocol to demonstrate child resistance, as well as developing changes in product formulation to reduce the toxicity of the detergent packet (Gaw et al. 2019; Hanway and Rodgers 2020). Additionally, Gaw et al. (2019) noted that individually wrapping laundry packets with child-resistant packing could mitigate risk, and that unit packaging does exist for at least one brand of dishwasher detergent packet. As ongoing mitigation strategies are being implemented by manufacturers, continued surveillance is needed to evaluate the effects and monitor the trends in detergent packet-related injuries and poisonings in Canada.

## Limitations

CHIRPP is a sentinel surveillance system, therefore, the injuries described in our study are not representative of all injuries in Canada, only those presenting to participating EDs. However, CHIRPP provides relatively good coverage of paediatric injury requiring ED care (Pickett et al. 2000). Nevertheless, certain groups are under-represented in the CHIRPP data, including rural inhabitants, Indigenous peoples including Inuit, Métis and First Nations, and fatal cases (Crain et al. 2016). Information is continuously entered into the CHIRPP database; therefore, some years do not yet have complete data. Future studies using additional data sources such as Canadian poison centre data and sales data may also help provide a more complete understanding of detergent packet-related poisonings and injuries in Canada.

## Conclusion

According to the CHIRPP database, children younger than five years of age accounted for the majority of detergent packet-related injuries and poisonings during the study period. Poisonings and eye injuries were most frequently reported. Overall, the number of detergent packet-related cases per 100,000 CHIRPP cases increased between 2012 and 2022. Although there was a decline in the number of detergent packet-related poisonings per 100,000 CHIRPP cases between 2015 and 2022, eye injuries increased between 2012 and 2022. Continued surveillance and prevention efforts are needed to reduce detergent packet-related injuries and poisonings in Canada, particularly among children and youth.

### Supplementary Information

Supplementary file 1.

Supplementary file 2.

Supplementary file 3.

Supplementary file 4.

Supplementary file 5.

## Data Availability

Data is available from the corresponding author on reasonable request.

## Abbreviations

AAPC: Average annual percent change
APC: Annual percent change
ASTM: American Society for Testing and Materials
CHIRPP: Canadian Hospitals Injury Reporting and Prevention Program
CI: Confidence interval
COVID-19: Coronavirus disease 2019
ED: Emergency department
NEISS: US National electronic injury surveillance system
NPDS: US National poison data system
