# Supplementary material for: Pediatric injuries and poisonings associated with detergent packets: results from the Canadian Hospitals Injury Reporting and Prevention Program (CHIRPP), 2011–2023
Source: Inj Epidemiol. 2024 Jul 11;11:31. doi: 10.1186/s40621-024-00513-5 (PMC11238368; doi:10.1186/s40621-024-00513-5)
Supplement: Supplementary file 1 [file 40621_2024_513_MOESM1_ESM.docx]

**Supplementary File 1.** Co-occurring injuries and poisonings among cases with multiple detergent packet-related injuries and poisonings, children and youth 17 years of age and younger, CHIRPP, April 1, 2011 - October 12, 2023 (n=54)

| **Multiple injuries** | **Count** | **Percent (%)** |
| --- | --- | --- |
| Poisoning and eye injury **ⁱ** | 27 | 50.0 |
| Multiple eye injuries **ⁱ** | 10 | 18.5 |
| Eye injury **ⁱ** and burn | 5 | 9.3 |
| Poisoning and burn | 3 | 5.6 |
| Multiple burns | 3 | 5.6 |
| Asphyxia and poisoning | 3 | 5.6 |
| Poisoning and internal caustic burn | 2 | 3.7 |
| Eye injury **ⁱ**, minor head injury, poisoning | 1 | 1.9 |
| **Total** | 54 | 100.0 |

Abbreviations: CHIRPP, Canadian Hospitals Injury Reporting and Prevention Program **ⁱ** "Eye injury" category includes globe only (including eye burn/corrosion) and foreign body in external eye.
