# Supplementary material for: Pediatric injuries and poisonings associated with detergent packets: results from the Canadian Hospitals Injury Reporting and Prevention Program (CHIRPP), 2011–2023
Source: Inj Epidemiol. 2024 Jul 11;11:31. doi: 10.1186/s40621-024-00513-5 (PMC11238368; doi:10.1186/s40621-024-00513-5)
Supplement: Supplementary file 2 [file 40621_2024_513_MOESM2_ESM.docx]

**Supplementary File 2.** Characteristics of laundry and dishwasher detergent packet-related cases, children and youth 17 years of age and younger, CHIRPP, April 1, 2011 - October 12, 2023 (n=869)

| **Characteristic** | **Laundry packet** | | **Dishwasher packet** | |
| --- | --- | --- | --- | --- |
|  | **Count** | **Column percent (%)** | **Count** | **Column percent (%)** |
| **Age group (years)** |  |  |  |  |
| < 2 | 262 | 35.8 | 107 | 78.1 |
| 2 to 4 | 362 | 49.5 | 20 | 14.6 |
| 5 to 9 | 67 | 9.2 | 4 | 2.9 |
| 10 to 17 | 41 | 5.6 | 6 | 4.4 |
| **Primary nature of injury** |  |  |  |  |
| Poisoning or toxic effect | 405 | 55.3 | 104 | 75.9 |
| Eye injury **ⁱ** | 256 | 35.0 | 12 | 8.8 |
| Other injury **ⁱⁱ** | 35 | 4.8 | 4 | 2.9 |
| No injury detected or not specified | 36 | 4.9 | 17 | 12.4 |
| **Total** | 732 | 100.0 | 137 | 100.0 |

Abbreviations: CHIRPP, Canadian Hospitals Injury Reporting and Prevention Program **ⁱ** "Eye injury" category includes globe only (including eye burn/corrosion) and foreign body in external eye.

**ⁱⁱ** "Other injury" category includes burn or corrosion, foreign body in respiratory/alimentary tract, asphyxia, internal caustic burn, superficial injury, foreign body in ear canal or soft tissue.
