# Supplementary material for: Pediatric injuries and poisonings associated with detergent packets: results from the Canadian Hospitals Injury Reporting and Prevention Program (CHIRPP), 2011–2023
Source: Inj Epidemiol. 2024 Jul 11;11:31. doi: 10.1186/s40621-024-00513-5 (PMC11238368; doi:10.1186/s40621-024-00513-5)
Supplement: Supplementary file 3 [file 40621_2024_513_MOESM3_ESM.docx]

**Supplementary File 3.** Characteristics of detergent packet-related injury and poisoning hospital admissions, children and youth 17 years of age and younger, CHIRPP, April 1, 2011 - October 12, 2023 (n=65)

| **Characteristic** | **Count** | **Percent (%)** |
| --- | --- | --- |
| **Sex** |  |  |
| Female | 20 | 30.8 |
| Male | 45 | 69.2 |
| **Age Group (years)** |  |  |
| < 2 | 40 | 61.5 |
| 2 to 4 | 16 | 24.6 |
| 5 to 9 | 1 | 1.5 |
| 10 to 17 | 8 | 12.3 |
| **Exposure mechanism** |  |  |
| Unintentional ingestion | 54 | 83.1 |
| Intentional ingestion | 8 | 12.3 |
| Bit into DP and contents got into eye/onto face or body | 2 | 3.1 |
| Squeezed/broke DP and contents got into eye/onto face or body | 1 | 1.5 |
| **Total** | 65 | 100.0 |

Abbreviations: CHIRPP, Canadian Hospitals Injury Reporting and Prevention Program; DP, detergent packet
