# Supplementary material for: Pediatric injuries and poisonings associated with detergent packets: results from the Canadian Hospitals Injury Reporting and Prevention Program (CHIRPP), 2011–2023
Source: Inj Epidemiol. 2024 Jul 11;11:31. doi: 10.1186/s40621-024-00513-5 (PMC11238368; doi:10.1186/s40621-024-00513-5)
Supplement: Supplementary file 4 [file 40621_2024_513_MOESM4_ESM.docx]

**Supplementary File 4.** Characteristics of intentional laundry detergent packet-related cases, children and youth aged 10 to 17 years, CHIRPP, April 1, 2011 - October 12, 2023 (n=22)

| **Characteristic** | **Count** | **Percent (%)** |
| --- | --- | --- |
| **Sex** |  |  |
| Female | 17 | 77.3 |
| Male | 5 | 22.7 |
| **Injury Year** |  |  |
| 2011 **ⁱ** to 2019 | 6 | 27.3 |
| 2020 to 2023 **ⁱ** | 16 | 72.7 |
| **Total** | 22 | 100.0 |

Abbreviations: CHIRPP, Canadian Hospitals Injury Reporting and Prevention Program **ⁱ** Data for years 2011 and 2023 do not include all months of the year.
Note: Information is continuously entered into the CHIRPP database; therefore, some years do not yet have complete data.
