# Supplementary material for: Pediatric injuries and poisonings associated with detergent packets: results from the Canadian Hospitals Injury Reporting and Prevention Program (CHIRPP), 2011–2023
Source: Inj Epidemiol. 2024 Jul 11;11:31. doi: 10.1186/s40621-024-00513-5 (PMC11238368; doi:10.1186/s40621-024-00513-5)
Supplement: Supplementary file 5 [file 40621_2024_513_MOESM5_ESM.docx]

**Supplementary File 5.** Detergent packet exposure mechanisms leading to an emergency department visit, children and youth 17 years of age and younger, by age group, CHIRPP, April 1, 2011 - October 12, 2023 (n=904)

| **Exposure mechanism** | **Age group (years)** | | | | | |
| --- | --- | --- | --- | --- | --- | --- |
|  | **0 to 4** | | **5 to 9** | | **10 to 17** | |
|  | **Count** | **Column percent (%)** | **Count** | **Column percent (%)** | **Count** | **Column percent (%)** |
| Unintentional ingestion | 497 | 63.6 | 16 | 21.3 | 1 | 2.1 |
| Squeezed/broke DP and contents got into eye/onto face or body | 214 | 27.4 | 55 | 73.3 | 23 | 48.9 |
| Bit into DP and contents got into eye/onto face or body | 34 | 4.3 | 2 | 2.7 | 0 | 0.0 |
| DP contents on hands and touched eye/face | 27 | 3.5 | 1 | 1.3 | 0 | 0.0 |
| Intentional ingestion | 0 | 0.0 | 0 | 0.0 | 22 | 46.8 |
| DP contents on bedding/clothing | 4 | 0.5 | 1 | 1.3 | 0 | 0.0 |
| Unknown or not further specified | 6 | 0.8 | 0 | 0.0 | 1 | 2.1 |
| **Total** | 782 | 100.0 | 75 | 100.0 | 47 | 100.0 |

Abbreviations: CHIRPP, Canadian Hospitals Injury Reporting and Prevention Program; DP, detergent packet
